# A randomized group antenatal care pilot showed increased partner communication and partner HIV testing during pregnancy in Malawi and Tanzania

**DOI:** 10.1186/s12884-021-04267-6

**Published:** 2021-11-24

**Authors:** Rohan D. Jeremiah, Dhruvi R. Patel, Ellen Chirwa, Esnath Kapito, Xiaohan Mei, Linda L. McCreary, Kathleen F. Norr, Li Liu, Crystal L. Patil

**Affiliations:** 1 College of Nursing, University of Illinois Chicago, Chicago, USA; 2 Kamuzu University of Health Sciences, Blantyre, Malawi; 3 School of Public Health, University of Illinois Chicago, Chicago, USA

**Keywords:** Antenatal care, Group healthcare, Maternal and newborn health, Partner communication, HIV testing, Sub-Saharan Africa

## Abstract

**Background:**

HIV testing at antenatal care (ANC) is critical to achieving zero new infections in sub-Saharan Africa. Although most women are tested at ANC, they remain at risk for HIV exposure and transmission to their infant when their partners are not tested. This study evaluates how an HIV-enhanced and Centering-based group ANC model-Group ANC+ that uses interactive learning to practice partner communication is associated with improvements in partner HIV testing during pregnancy.

**Methods:**

A randomized pilot study conducted in Malawi and Tanzania found multiple positive outcomes for pregnant women (*n* = 218) assigned to Group ANC+ versus individual ANC. This analysis adds previously unpublished results for two late pregnancy outcomes: communication with partner about three reproductive health topics (safer sex, HIV testing, and family planning) and partner HIV testing since the first antenatal care visit. Multivariate logistic regression models were used to assess the effect of type of ANC on partner communication and partner testing. We also conducted a mediation analysis to assess whether partner communication mediated the effect of type of care on partner HIV testing.

**Results:**

Nearly 70% of women in Group ANC+ reported communicating about reproductive health with their partner, compared to 45% of women in individual ANC. After controlling for significant covariates, women in group ANC were twice as likely as those in individual ANC to report that their partner got an HIV test (OR 1.99; 95% CI: 1.08, 3.66). The positive effect of the Group ANC + model on partner HIV testing was fully mediated by increased partner communication.

**Conclusions:**

HIV prevention was included in group ANC health promotion without compromising services and coverage of standard ANC topics, demonstrating that local high-priority health promotion needs can be integrated into ANC using a Group ANC+. These findings provide evidence that greater partner communication can promote healthy reproductive behaviors, including HIV prevention. Additional research is needed to understand the processes by which group ANC allowed women to discuss sensitive topics with partners and how these communications led to partner HIV testing.

## Contributions

- HIV-enhanced and Centering-based group antenatal care substantially increased partner communication about reproductive health and partner HIV testing without compromising requisite ANC health promotion content.
- HIV enhancement included HIV transmission and testing information, an “exchange game” and role plays practicing partner communication about sensitive topics.
- The impact of this type of antenatal care on partner HIV testing was fully mediated by increased partner communication, providing further evidence that improved partner communication promotes healthy reproductive behaviors for couples.
- The group model of care offers an innovative approach to address diverse health concerns and target populations, including engaging men in primary healthcare

## Background

In sub-Saharan Africa, where two-thirds of new HIV infections occur, routine HIV testing of pregnant women at antenatal care (ANC) and treatment for those who test positive is nearly universal [1]. HIV testing at ANC remains a critical strategy to achieve zero new infections by 2030 [2–5]. However, women testing negative at their first ANC visit remain at risk for new infection as do their infants [6, 7].

Even though testing of partners at ANC would help with risk reduction decisions [6], efforts to bring men into ANC have had limited success in sub-Saharan Africa [8, 9]. Providing HIV self-testing (HIVST) to women at ANC for their partner is another strategy that has been implemented in Ugand and Malawi [10, 11]. In Malawi, providing an HIVST alone (i.e., without cash incentives) did not improve partner testing outcomes [6]. Cultural and structural barriers, including gender and age inequalities, norms against discussing sexuality, employment demands, poverty, and transportation difficulties, hinder HIV testing for male partners at ANC [12–15].

The failure to successfully engage men in ANC shifts the burden of getting partners tested to pregnant women. Frank discussions initiated by women generally contradict family and relationship norms and are associated with mistrust and interpersonal violence [16, 17]. Therefore, equipping pregnant women with communication skills that might allow them to safely and effectively engage with partners in discussing sensitive topics like HIV testing and condom use are needed. HIV prevention interventions show that when communication skills are strengthened, partner communication increases and there is an adoption of risk reduction behaviors, including condom use [18–20]. Currently, in sub-Saharan Africa, partner communication is not part of the ANC health promotion content. During each visit, women may be present for a lecture that provides a rapid overview of important ANC-related health promotion topics before meeting with a midwife individually for a brief physical assessment. Lectures do not provide opportunities for in-depth discussion or skill building.

Group ANC is an innovative alternative to individual ANC that allows for extended health promotion discussion and skill building. The group ANC model, CenteringPregnancy, was developed and tested in the US and has strong evidence of effectiveness [21–23]. Its approach is based on a consistent group of 8-12 women at a similar pregnancy stage attending all of their 2-h visits together. Each visit includes health assessments, interactive learning for health promotion, and opportunities to socialize and build a sense of community [23]. Centering-based group ANC is associated with positive outcomes including declines in prematurity rates and improved attendance, satisfaction, breastfeeding practices, and feasibility of bringing it to scale [22–26]. When HIV and STI prevention content were integrated with CenteringPregnancy (CP+), there were improvements in safer sex behaviors and family planning uptake [21, 27].

To determine whether the Group ANC+ model benefits women in sub-Saharan Africa where HIV prevalence is high, we adapted this model for use in Malawi and Tanzania [28]. We incorporated partner communication activities from a peer group intervention for HIV prevention intervention [29–31]. Previously published outcomes of this randomized pilot showed that more women in group ANC reported receiving essential services such as measuring blood pressure and discussion of more ANC health promotion topics. They also had increased ANC and postpartum attendance, satisfaction with ANC, HIV prevention knowledge, more pregnancy-related empowerment, and more comprehensive care as measured by services and educational topics [32–34].

In this study, we examined unpublished data from our pilot to evaluate the effect of type of ANC (group or individual) on partner communication and partner HIV testing outcomes during the current pregnancy. We then examined whether partner communication mediated the relationship of type of ANC care on partner testing.

## Methods

### Design

We use data from a 2-arm randomized pilot study conducted in Malawi and Tanzania that compared outcomes for pregnant women randomly assigned to individual or Group ANC+. Prior to enrollment, computer assigned random assignment slips representing each arm of the study were placed in identical envelops and manually shuffled to randomized order. After completing the baseline survey, the woman selected the first envelope in the batch which revealed the assignment to the woman and study team. Previously published work describes details about the randomization process, retention rates, methods, and primary outcomes [32].Before data collection, we received necessary approvals from three institutional review boards, the College of Medicine Research and Ethics Committee in Malawi, the National Institute for Medical Research in Tanzania, and the University of Illinois Chicago. We also received approval from the Ministries of Health and administrators at participating clinics.

### Setting and sample

Malawi and Tanzania are low-income sub-Saharan African countries with high rates of maternal and infant morbidities and mortality. This pilot was launched in 2014 in two rural clinics in central Malawi and one urban clinic in Dar es Salaam, Tanzania where ANC followed focused antenatal care guidelines for four visits [35]. Women over the age of 15, with a gestational age between 20 and 24 weeks were recruited for participation. After completing the informed consent process, pregnant women completed the baseline survey and then were randomly assigned to one of two study conditions [32]. Participants were compensated with the equivalent of US$5 for taking the surveys. The compensation was not linked to their level of care engagement. A consort diagram with detailed recruitment and retention was previously published [32].

### Study conditions

#### Individual ANC (control)

Services are provided on a first-come, first-served basis. At all visits, women met with a midwife individually for a brief physical assessment. Laboratory tests (including HIV testing) were undertaken at their first visit. Although not required, often women are present for a health lecture that provides a rapid overview of important topics. Women were expected to complete four visits and return to the clinic for two postnatal visits at one and 6 weeks after delivery. Attendance is recorded, but there is no reminder system in place.

#### Group ANC+ (intervention)

Women had the same number of scheduled visits as women in the individual arm. However, after an individual first (intake) visit, the other ANC visits and their 6-week postnatal check-ups occurred with the same consistent group of women with an approximately similar expected delivery date. Each scheduled 2-h appointment included women’s self-measurement and recording of their blood pressure and weight, followed by a one-on-one physical assessment in a group space with the midwife. The group then gathered in a circle, and a trained midwife and assistant facilitated interactive educational health promotion activities and discussions that focused on partner communication and HIV testing. Opportunities for community building occur throughout the session.

### Measures

#### Variable of interest

The randomization indicator, Type of ANC (individual ANC or Group ANC+), was the primary variable of interest.

#### Dependent variables

Partner communication was measured by asking women whether they discussed three sexual health topics with their partner since coming to ANC: safer sex, HIV testing, and family planning. These three items were combined to produce the total number of items discussed, possible range, 0-3. Partner HIV testing was measured by asking women whether their partner had an HIV test since the woman started coming to ANC.

#### Covariates

Covariates included age at baseline (< 20, 20-34, 35+), gravidity (1 or > 1), education category (less than primary school, completed primary school, or more than primary school), relationship status (whether married or living with a partner, coded yes or no), parity (0 or ≥ 1), religion (Christian or Muslim), and access to an independent source of income (yes or no). We also included the country (Malawi or Tanzania) as a covariate because it encapsulates many economic and sociodemographic differences.

### Procedure

Women completed a survey in late pregnancy (third trimester) that included the partner communication and HIV testing questions. Of the 218 women enrolled at baseline, 88% completed the late pregnancy survey. Women assigned to individual ANC in Malawi had the lowest retention rate (40/58 [69.0%]).

### Analysis

**Fig. 1:**
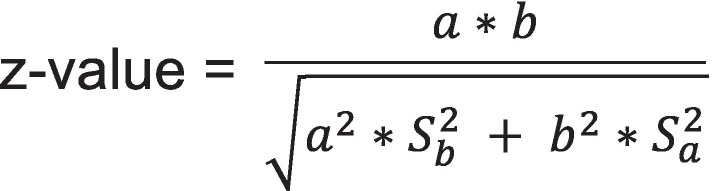
Sobel test for mediation effect [37]

Bivariate relationships were examined between the type of ANC and partner communication and partner HIV testing using Chi-squared tests. We then used the mediation analysis process using the procedures described by Baron and Kenny (1986) [36], which requires establishing that: (1) the causal variable (the type of ANC) significantly affected the outcome (partner HIV testing); (2) the causal variable also significantly affected the mediator (partner communication); and (3) when the mediator is added to the regression model, the relationship between the causal variable and outcome is no longer significant. We used multivariate logistic regression and cumulative ordinal regression models to examine the impact of the type of ANC on partner HIV testing and communication, respectively. Stepwise model selection method was employed in these regression models so that the estimates of the type of ANC effects were adjusted for significant covariates. We then introduced partner communication as a predictor of partner HIV testing to examine whether this variable mediated the effect of type of ANC on the relationship. Effect sizes from all logistic regression models were reported using Odds Ratios (OR) with 95% confidence interval estimates. The indirect effect of type of ANC on partner testing outcome through partner communication was calculated and tested using the Sobel method (1982) [37] given in the formula as follows, where *S*_*a*_ and *S*_*b*_ refered to the standard errors of the effects *a* and *b* in the mediation process (Fig. 1). All statistical tests were two-sided, controlled for Type I error probability of 0.05.

## Results

A full description of women’s obstetric and sociodemographic characteristics for the entire sample and by country is published elsewhere [32, 33]. In brief, about half of the participants were recruited in Malawi and half in Tanzania (*n* = 218; 112 in Malawi and 106 in Tanzania). Women were, on average, 27 years old and 14% were under the age of 20. One-third were primiparous. Nearly all women (91.6%) reported being in a relationship. However, random assignment resulted in 13 of the 18 women who were not in a relationship being assigned to Group ANC+, a significance difference. Most women (69.8%) reported having an independent income source such as a job, farming, or selling goods. The majority (74.9%) had 8 years or less of education. 76.3% identified as Christian and 23.7% were Muslim.

Type of ANC was associated with greater partner communication (Table 1). Nearly 70% of women in group ANC reported that they had discussed all three topics, compared to 45% of women in individual ANC. More women (54.8%) from group ANC reported that their partner got an HIV test compared to those in individual ANC (45.2%) (*p*-value = 0.078).

**Table 1:** The effect of type of ANC on partner communication and HIV testing

	Group (n/%)	Individual (n/%)	***p***-value
**Number of topics discussed**			
0	5 (4.81)	15 (17.05)	0.0008***
1	3 (2.88)	10 (11.36)	
2	24 (23.08)	23 (26.14)	
3	72 (69.23)	40 (45.45)	
**Partner, HIV test**			
Yes	57 (54.81)	47 45.19)	0.0780
No	37 (42.05)	51 (57.95)	

The results of the three regression models associated with the mediation analysis relationships among type of ANC, partner community and partner HIV testing are summarized in Table 2. Model 1 estimates the *total effect* (*c*) of the causal variable on the outcome of partner HIV testing without taking the mediator into account. Only two covariates (country and whether married or living with a partner) were significantly associated with partner testing. More partner tests were reported in Malawi than in Tanzania (Tanzania vs. Malawi, OR 2.38; 95% CI: 1.28, 4.43). Women who were married or living with their partner were four times more likely to say that their partner had been tested during this pregnancy (OR 4.24; 95% CI: 1.31, 13.72). After controlling for significant covariates, women in group ANC were twice as likely as those in individual ANC to report that their partner got an HIV test (OR 1.99; 95% CI: 1.08, 3.66).

**Table 2:** Results of mediation analysis testing whether partner communication mediates the effect of type of ANC on partner HIV testing

	Non-mediation model		Mediation Models			
	Model 1. Effect of Type of ANC on Partner HIV Testing (*c*)		Model 2. Effect of Type of ANC on Partner Communication (*a*)		Model 3. Effect of Partner Communication (*b*) and Type of ANC on Partner HIV Testing (*c* ′ )	
	Estimate (SE)	OR (CI)	Estimate (SE)	OR (CI)	Estimate (SE)	OR (CI)
Partner Communication, late pregnancy					0.45* (0.18) ^*b*^	1.58 (1.10, 2.26)
Type of ANC (Group vs Individual [ref])	0.69** (0.31)^*c*^	1.99 (1.08, 3.66)	1.43*** (0.32)^*a*^	4.17 (2.22, 7.83)	0.44 (0.33)^*c’*^	1.55 (0.81, 2.95)
**Co-variates**						
Country (Tanzania vs Malawi [ref])	0.87* (0.32)	2.38 (1.28, 4.43)	1.73*** (0.34)	5.67 (2.93, 10.95)	0.58 (0.34)	1.79 (0.92, 3.47)
Relationship Status (if married or living with partner vs not in relationship [ref])	1.44* (0.60)	4.24 (1.31, 13.72)	0.87 (0.60)	2.39 (0.74, 7.69)	1.36* (0.61)	3.90 (1.18, 12.83)

*p*-values: < 0.05 * < 0.01 ** < 0.001 ***​

Note: Indirect Effect: *ab* = 1.43*0.45 = 0.65; Sobel Test for Indirect Effect: SE = 0.30, z statistics = 2.16, *p*-value = 0.03

Model 2 estimates the effect of type of ANC on partner communication, which is part of the *indirect effect* of Type of ANC on HIV testing through communication. The analysis controlled for the same covariates that related to partner HIV testing, country, and relationship status. Country was also a significant predictor of communication, with individuals in Tanzania communicating with their partner significantly more than those in Malawi (OR 5.67, 95% CI: 2.93-10.95); but relationship status was not significantly associated with communication. After controlling for covariates, women in group ANC were over four times more likely than women in individual care to report more communication items with their partners (OR 4.19, 95% CI: 2.22, 7.83).

In Model 3, we added partner communication to test whether it mediated the relationship between the type of ANC and partner HIV testing. In this regression model, partner communication significantly related to partner HIV testing (OR 1.58, 95% CI: 1.10, 2.26), forming a significant indirect effect from Type of ANC on partner HIV testing through partner communication. In this model, the type of care is no longer a significant predictor of partner HIV testing. Relationship status also positively relates to partner HIV testing in this model, but country is no longer significant.

**Fig. 2:**
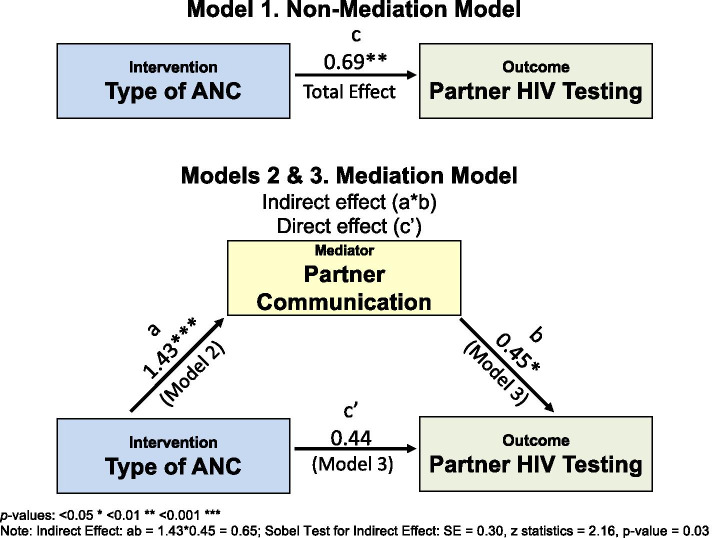
Mediation effect of Type of ANC on Partner HIV Testing through Partner Communication. *p*-values: < 0.05 * < 0.01 ** < 0.001 ***. Note: Indirect Effect: ab = 1.43*0.45 = 0.65; Sobel Test for Indirect Effect: SE = 0.30, z statistics = 2.16, *p*-value = 0.03

Figure 2 shows the *direct effect* of type of care on Partner HIV Testing (*c”*) and the *indirect effect*, which consists of the effect of the intervention on the mediator (*a*) and the effect of the mediator on the outcome (*b*).The estimate of the indirect effect was obtained by taking the product of *ab*, and the standard error and inference of the indirect effect were obtained using the Sobel method (1982) [37]. As indicated by Judd and Kenny (1981) [38] and Baron and Kenny (1986) [36], if the total effect (*c*) and the indirect effect (*ab*) were significant, and the direct effect of (*c”* becomes non-significant with the inclusion of an indirect path, then *complete mediation* has occurred. Using the Sobel method (1982) [37], the indirect effect of type of ANC on the outcome (partner testing) through the mediator (partner communication) was statistically significant (estimate = 0.65, SE = 0.30, *p*-value = 0.03).

## Discussion

Our Group ANC+ model is the only such model being implemented in sub-SaharanAfrica, where about two-thirds of all global new HIV infections occur [28, 32]. Group ANC +, which allows for time group-based learning and practice, was associated with both increased partner communication about important reproductive health topics and partner HIV testing during pregnancy. As shown by our previous studies [32, 34], these HIV prevention-related outcomes occurred without jeopardizing coverage of required ANC health promotion topics or other maternal and child health ANC benefits. If group ANC models are being planned or implemented in high-HIV prevalence African countries [39–42] or other regions might consider incorporating the HIV prevention content that HIV negative women so urgently need.

Group ANC+ provides a tested example showing how group ANC can effectively incorporate a local high-priority health promotion context that may not otherwise be covered in standard ANC. Because each two-hour Group ANC+ visit allocates 60-75 min for interactive health promotion, this extended time allows incorporating important local health priorities beyond those typically addressed in ANC. This adaptation showed evidence of reaching male partners indirectly which expands the impact of ANC on family health. Integrating a mental health focus into group ANC in Mali is another example illustrating the flexibility of the group ANC model [42]. ANC is a well-established and trusted health service that offers a gateway to reach pregnant women and their partners indirectly. Therefore, integrating local high priority health promotion topics into ANC is likely to have a significant public health impact on maternal, infant, and family health outcomes.

This study provides further evidence about the importance of partner communication as a factor that plays a critical role in promoting healthy behaviors [43]. Results showed that partner communication mediated the effect that Group ANC+ had on partner HIV testing; when communication between pregnant women and their partners increased, so did their partner’s testing. These findings are congruent with previous research linking partner communication with risk reduction for sexual behaviors, including condom use [19, 20, 44, 45]. Additional research is needed to describe the processes by which Group ANC+ led women to discuss these sensitive topics with their partners, when and how women brought up those conversations, partners’ responses, and how their communications resulted in partner testing.

Group care provides an alternative health care delivery model to address multiple health needs and reach target populations. The model’s three core components, health assessement, interactive learning, and community building are not specific to ANC. The model has been used for parenting (incorporating both well-woman and well-child care) and for diseases requiring self-management skills including diabetes and sickle cell disease in the US [46–48]. However, there are few examples of group care models beyond group ANC in middle and low resource settings. Group care might provide an innovative strategy for engaging men in health promotion throughout the life course. This global healthcare gap persists in nearly all countries regardless of resources and health system factors.

### Limitations

This study has several limitations. As a pilot, the sample was relatively small, and retention was suboptimal for women in one country’s control group. Although we included urban and rural sites, we did not have both types of settings in both countries. A second limitation is the reliance on women’s reports for most measures. Given the limited scope of the study, no other another data sources were used to corroborate women’s reportings. Therefore, it is possible that women’s responses about communication were influenced by social desirability bias for those who attended group care because the interactive activities promoted improving communication skills and knowing one’s partner status. Third, random assignment resulted in significant differences in relationship status by study condition. However, only eighteen women reported not being in a relationship. Given these small numbers, it is unlikely that the differences in relationship status by study condition affected our primary results. Fourth, we did not systematically assess partner communication skill building beyond having a discussion or not; therefore, we cannot draw conclusions about the holistics impact these discussions had on relationships. Last, we did not have repeated measures for HIV testing and partner communication. In our cross-sectional analysis, we based our assumption about the direction of effect from previously published studies linking partner communication to better health outcomes [19, 20, 44, 45].

### Implications

This study shows that group ANC can be enhanced with HIV-prevention content without compromising other health promotion content in sub-Saharan Africa. Group ANC+ effectively increased partner communication about sensitive reproductive health issues, which led to more partner HIV testing in a region where HIV incidence is high. HIV prevention-enhanced group ANC can make a significant public health contribution throughout sub-Saharan Africa and other high HIV-prevalence regions. Group ANC has been expanded successfully in the US and can be extended for diverse health concerns and target populations globally, including engaging men in primary healthcare with implications for improving family health.

## Data Availability

De-identified survey data will be made available by emailing a request to: rjerem@uic.edu.
